# Castration-resistant prostate cancer monitoring by cell-free circulating biomarkers

**DOI:** 10.3389/fonc.2024.1394292

**Published:** 2024-09-10

**Authors:** Eva Chrenková, Hana Študentová, Kateřina Holá, Zuzana Kahounová, Romana Hendrychová, Karel Souček, Jan Bouchal

**Affiliations:** ^1^ Department of Clinical and Molecular Pathology, Institute of Molecular and Translational Medicine, Faculty of Medicine and Dentistry, Palacký University and University Hospital, Olomouc, Czechia; ^2^ Department of Oncology, Faculty of Medicine and Dentistry, Palacký University and University Hospital, Olomouc, Czechia; ^3^ Department of Cytokinetics, Institute of Biophysics of the Czech Academy of Sciences, Brno, Czechia

**Keywords:** castration-resistant prostate cancer, liquid biopsy, biomarker, progression monitoring, therapy response

## Abstract

**Background:**

Prostate cancer is the second leading cause of male cancer-related deaths in Western countries, which is predominantly attributed to the metastatic castration-resistant stage of the disease (CRPC). There is an urgent need for better prognostic and predictive biomarkers, particularly for androgen receptor targeted agents and taxanes.

**Methods:**

We have searched the PubMed database for original articles and meta-analyses providing information on blood-based markers for castration-resistant prostate cancer monitoring, risk group stratification and prediction of therapy response.

**Results:**

The molecular markers are discussed along with the standard clinical parameters, such as prostate specific antigen, lactate dehydrogenase or C-reactive protein. Androgen receptor (AR) alterations are commonly associated with progression to CRPC. These include amplification of AR and its enhancer, point mutations and splice variants. Among DNA methylations, a novel 5-hydroxymethylcytosine activation marker of TOP2A and EZH2 has been identified for the aggressive disease. miR-375 is currently the most promising candidate among non-coding RNAs and sphingolipid analysis has recently emerged as a novel approach.

**Conclusions:**

The promising biomarkers have the potential to improve the care of metastatic prostate cancer patients, however, they need further validation for routine implementation.

## 1 Introduction

**Figure 1:**
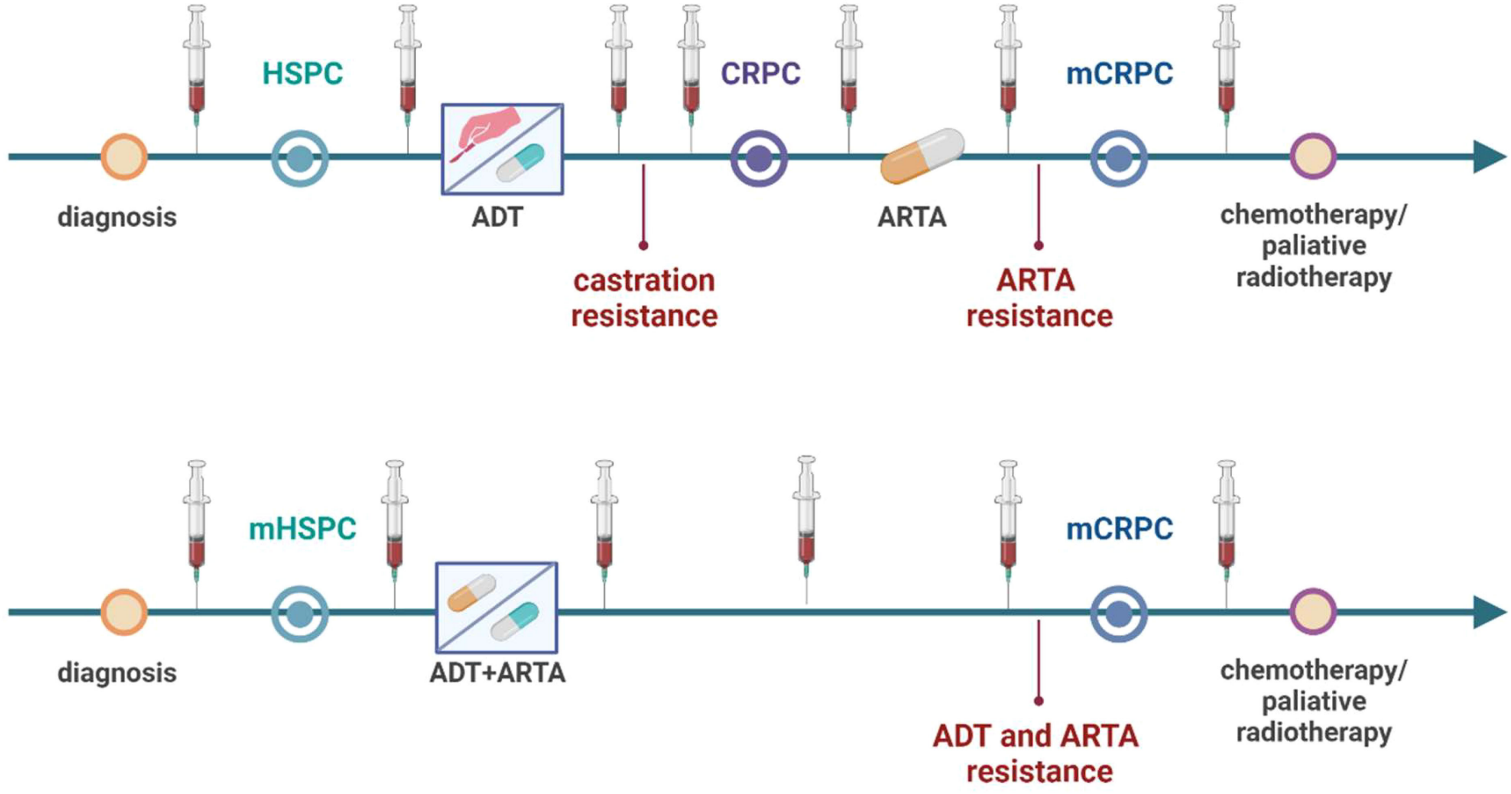
Scheme of liquid biopsy sampling in relation to different treatment schemes in hormone-sensitive prostate cancer (HSPC) and castration-resistant prostate cancer patients (CRPC). HSPC patients are first treated with androgen-deprivation therapy (ADT) and androgen-receptor targeted agents (ARTA) or androgen-receptor pathway inhibitors (ARPI) are used after castration resistance occurrence leading to ARTA resistance and metastatic CRPC (mCRPC). Now, based on novel treatment protocol metastatic HSPC patients treated with ADT and ARTA combination leading to double-resistance and mCRPC. Liquid biopsy sampling can be performed at different time points throughout the course of treatment for monitoring of progression and metastatic activity of the cancer. Created with BioRender.com.

Prostate cancer (PC) is the most common malignancy and the second leading cause of male cancer related deaths in developed countries (1). PC begins to grow as an asymptomatic localized cancer. However, PC is often diagnosed in the higher stage when it continues to grow through the prostate envelope and becomes advanced cancer, making treatment more challenging. While prostatectomy is an option for localized or locally advanced PC, androgen-deprivation therapy (ADT) is commonly employed for metastatic disease (**Figure 1**).

PC cells are often androgen-dependent and need androgen stimuli for proliferation. Therefore, ADT aims to reduce androgen concentration and inhibit PC cell growth. Over time, castration-resistant PC (CRPC) can emerge, showing resistance to ADT. Some androgens can still be produced by the adrenal glands, fuelling PC growth, and AR-pathway inhibitors (ARPI) are used to neutralize this androgen effect by acting as androgen antagonists (e.g., enzalutamide) or further inhibit androgen production (e.g., abiraterone). ARPI are also known as AR-targeting agents (ARTA) (2).

**Figure 2:**
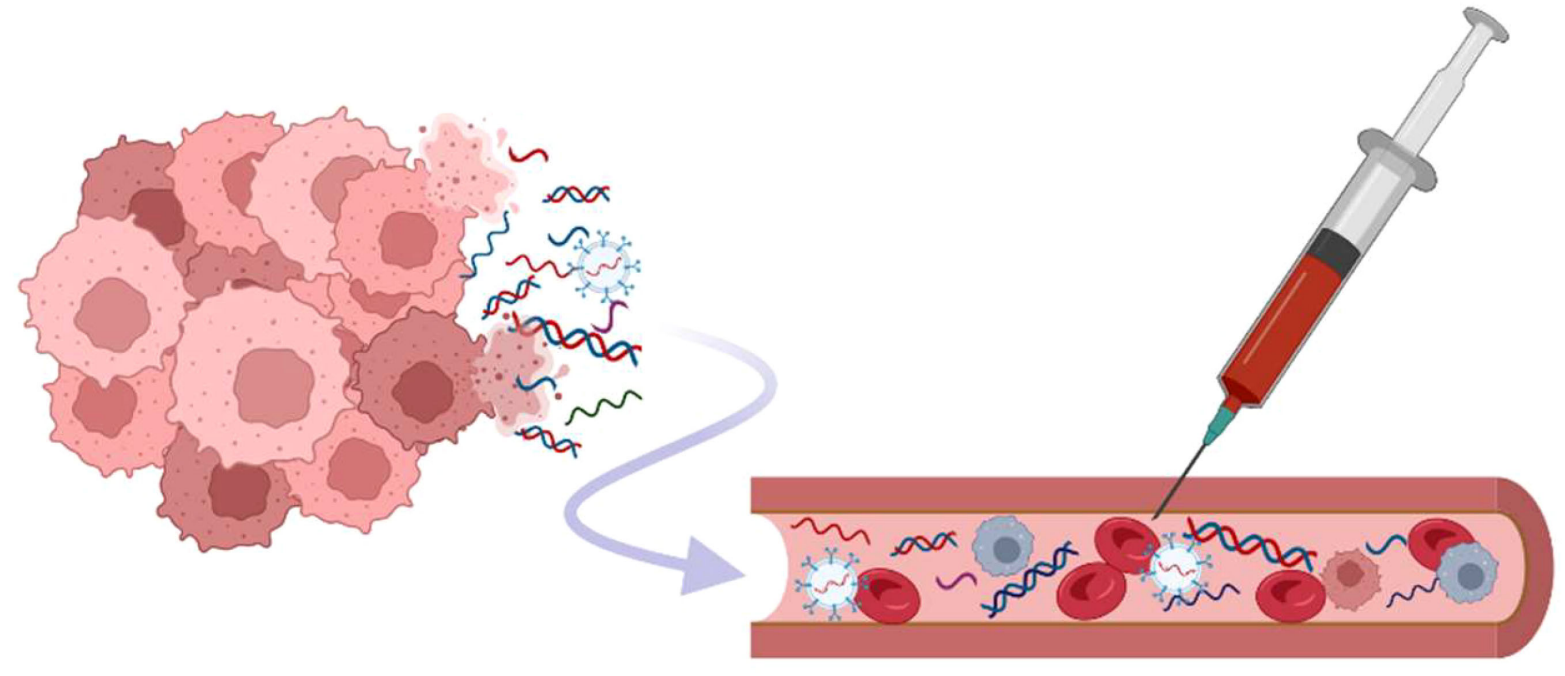
Different types of biomarkers are present in circulation. The cell-free DNAs, various types of RNAs, as well as nucleic acid captured in exosomes, are released from cancer cells in different pathological or normal biological processes (apoptosis, necrosis, tissue damage, …). There is a need for standardization of both the acquisition of samples and validation of methods used for the analysis to ensure the reproducibility of analysis and the accuracy of the results (measured in one workspace or across laboratories). Created with BioRender.com.

The therapeutic armamentarium for treating metastatic CRPC (mCRPC) in clinical practice has evolved in recent decades. Nowadays, there are several classes of therapeutic options to manage the disease progression. Besides chemotherapeutic agents such as docetaxel and cabazitaxel, ARPI, innovative radioligand therapy represented by Lu-PSMA, and PARP inhibitors have extended the therapeutic scheme. Nevertheless, the specific biomarkers to choose the best therapeutic approach are still challenging to find because of the heterogeneity of CRPC (3, 4). Therefore, the liquid biopsy (LB) can offer a tremendous predictive tool for future personalized approaches, providing varied material for molecular examinations (**Figure 2**). Recent reviews were primarily dedicated to circulating tumor cells (5–8), and we have also focused on their potential in modeling metastatic PC (9). It’s worth noting that urine testing also holds great potential for examining PC patients; for more detailed information about LB based on urine examination, see the dedicated review (10). This article aims to present circulating cell-free markers with potential value in progression monitoring of CRPC from blood samples, including whole blood, plasma, or serum. Importantly, novel markers should be evaluated in the context of the established clinical protocols and currently available tools.

## 2 Prognostic biomarkers for risk group stratification from a perspective of clinical practice

The current decision-making by the physicians is typically based on metastasis sites (nodes, bones, visceral), the progression pace, and the previous road of the treatment (3). Biomarkers extensively studied as a prognostic factor in patients with mCRPC are inflammatory response cells. The most valuable benefit of these markers is their easy accessibility in every clinical laboratory, cost-effectiveness, the possibility to evaluate them retrospectively, and their known prognostic correlation with many malignancies. Altered infiltration of the tumor microenvironment with various subgroups of lymphocytes is often associated with a prognosis and inflammation-promoted progression of the malignancy. The most used inflammation-related indicators are the neutrophil-to-lymphocyte ratio (NLR), platelet-to-lymphocyte ratio (PLR), and C-reactive protein (CRP).

In several meta-analyses, it was shown that higher NLR (cut-off value >3) correlates with poor overall survival (OS) in patients with advanced and metastatic PC treated with abiraterone or enzalutamide (11, 12). Recent reviews dedicated to cells of inflammation and their prognostic role in PC can be found elsewhere (13). Just for demonstration, Lolli et al. published a retrospective study with 230 patients diagnosed with mCRPC treated with abiraterone. The patients with NLR higher than three prior abiraterone had 14.7 months median OS, and those with NLR lower than 3 had 20.4 months median OS (14). Similar conclusions were also reported in a study by Koo and coworkers. They performed a retrospective analysis of 303 mCRPC patients treated with docetaxel before/after ARPI therapy. The NLR cut-off value of 2.5 was used for the stratification. The patients with NLR lower than 2.5 had better cancer-specific survival (CSS). Hence, this group (NLR<2.5) profited from the treatment sequence docetaxel-to-ARPI with better progression-free survival (PFS) and CSS than the group with the opposite treatment sequence (15).

PLR is another inflammation-related biomarker often reported with NLR as a systemic immune-inflammation index (SII). This index overall reflects the immune status connected with prognosis as the increased platelets can play an active role in the development of distant bone metastasis (16), while tumor-related neutrophils promote tumor growth and progression (17). On the other hand, lymphocytes have an anti-tumor role connected with apoptosis and proliferation suppression (17). Thus, the SII reflects the infiltration of the tumor microenvironment with immune cells that will determine further progression. The most used cut-off value for stratification is 535; patients with higher values have worse median OS and a more significant hazard ratio (18). The inflammatory response cells, therefore, can efficiently serve as effective biomarkers for prognosis prediction for patients with mCRPC.

CRP and procalcitonin (PCT), another non-specific inflammation-related indicator, have been associated with any inflammation within the body. They are usually increased in case of systemic infection or tissue damage but have also been described as reflecting the prognosis of cancer. In PC patients, the role of CRP has so far been inconclusive. Elevated preoperative CRP (CRP≥5mg/L) correlated with a postoperative pathological diagnosis of PC with aggressive patterns (19). Similarly, CRP levels before prostate biopsy were associated with increased Gleason score (20). Regarding prognosis, the higher levels of CRP were assigned as an independent predictor of poor OS in mCRPC (21). On the contrary, other studies claimed the absence of any correlation between CRP and PC (22, 23). PCT was helpful in terms of PC diagnostic accuracy since PCT was demonstrated to correlate with PC development (24, 25).

As an immune-related biomarker, CRP is often used in multivariable analysis to boost the significance of mCRPC stratification. The Glasgow Prognostic Score (GPS), used to determine cancer outcomes in general, is worth mentioning. The value of CRP and albumin from this prognostic score can even be explicitly refined for mCRPC diagnosis as was done in the work of Ando et al. in 2021. They set the cut-off value of CRP to 3 mg/L and albumin to 35 g/L to get to the high-sensitivity modified Glasgow prognostic score (26). These values were used for stratification of mCRPC patients treated with docetaxel; the clinical significance of this score can be seen in **Table 1**. A better result was reached when this score was combined with starting prostate-specific antigen (PSA) and testosterone levels (**Table 1**).

**Table 1:** Prognostic stratification of metastatic castration-resistant prostate cancer (mCRPC) patients based on clinical biomarkers and various cancer and health-related factors.

cohort and treatment	stratification predictors (score: 0 or 1)	risk groups (the sum score of predictors)	median OS	reference
211 mCRPC patients ARPI/docetaxel	Time of ADT 0: ADT≥12 M 1: ADT<12 M ALP 0: ALP ≤ 350 U/dL 1: ALP>350 U/dL Hemoglobin 0: Hb>110 mg/L 1: Hb ≤ 110 mg/L	Low risk (0) Intermed. risk (1) Poor risk (≥2)	NR*(>60 months) 27 months 12 months	Uchimoto et al., 2021 (160)
131 CRPC patients docetaxel	High-sensitivity modified Glasgow prognostic score 0: CRP<3 mg/L + albumin≥35 g/L 1: CRP≥3 mg/L + albumin≥35 g/L 2: CRP≥3 mg/L + albumin<35 g/L	Low risk (0) Intermed. risk (1) Poor risk (≥2)	51.8 months 18.1 months 8.5 months	Ando et al., 2021 (26)
131 CRPC patients docetaxel	HS-m Glas. prognostic score 0: CRP<3 mg/L + albumin≥35 g/L 1: CRP≥3 mg/L + albumin≥35 g/L 2: CRP≥3 mg/L + albumin<35 g/L PSA level 0: PSA<28.9 mg/mL 1: PSA≥28.9 mg/mL Testosterone 0: TST<130 ng/L 1: TST≥130 ng/L	Low risk (0-1) Intermed. risk (2-3) Poor risk (4)	58.3 months 21.2 months 5.4 months	Ando et al., 2021 (26)
196 mCRPC patients ARPI/docetaxel/ cabazitaxel	dNLR 0: dNLR<1.51 1: dNLR≥1.51 Lactate dehydrogenase 0: LDH≤ULN 1: LDH>ULN	Low risk (0) Intermed. risk (1) Poor risk (2)	46.2 months 28.9 months 16.6 months	Yamada et al., 2020 (161)
45 mCRPC patients cabazitaxel	PSA level 0: PSA ≤ 100 ng/mL 1: PSA>100 ng/mL Abs. monocyte count 0: AMC<400 per µL 1: AMC≥400 per µL Visceral metastases 0: NO 1: YES	Low risk (0) Intermed. risk (1-2) Poor risk (3)	23.3 months 16.1 months 2.5 months	Kosaka et al., 2018 (162)
519 mCRPC patients ^223^RaCl_2_	dNLR 0: dNLR<3.1 3: dNLR≥3.1 ECOG-PS 0: 0-1 1: 2-3 N bone metastases 0: <6 1: 6-20 3: >20 ALP 0: <220 U/L 2: ≥220 U/L PSA 0: <44 ng/mL 2: ≥44 ng/mL	Low risk (0-2) Intermed. risk (3-4) Poor risk (5-10)	31 months 26.6 months 9.6 months	Bauckneht et al., 2022 (163)
39 mCRPC patients II. line enzulatamide	CgA <120 CgA 120-360 CgA ≥360	Low risk Intermed. risk Poor risk	>12.2 months 9.4 months 3.4 months	Conteduca et al., 2014 (43)

ADT (androgen deprivation therapy), ALP (alkaline phosphatase), AMC (absolute monocyte count), ARPI (AR-pathway inhibitors), CgA - chromogranin A, CRP (C-reactive protein), dNLR (derived neutrophil-to-lymphocyte ratio), ECOG-PS (Eastern Cooperative Oncology Group Performance Status), Hb (hemoglobin), LDH (lactate dehydrogenase), NR* (not reached), TST (testosterone), ULN (upper limit of normal), OS (overall survival).

PSA is often used as a monitoring tool for the progression of the mCRPC disease. According to the Response Evaluation Criteria in Solid Tumors (RECIST) version 1.1, the progression of PC is defined as three consecutive elevated values of PSA resulting in two 50% rises over the nadir or the appearance of ≥2 bone lesions on bone scan or soft tissue lesions enlargement on computed tomography (CT). Nevertheless, the level of PSA and PSA kinetic are treatment-sensitive parameters. For example, non-rising PSA with metastatic radiographic progression is often observed in mCRPC patients treated with enzalutamide (27). In combination with the heterogeneity of the disease, the non-rising PSA can lead to delayed detection of the progressing disease. Similarly, initial PSA and mainly PSA response (PSAr; ≥50% decrease) as stratification factors must be used cautiously and only in cohorts with the same type of treatment. Still, the PSA kinetic (PSA NADIR, time to NADIR) during the initial ADT (during metastatic hormone-sensitive PC) can be helpful prognostic factors for patients with further PC progression to mCRPC. For example, in a study by Hamano and coworkers, the patients with PSA nadir lower than 0.64 ng/mL in less than seven months after the start of ADT had better OS after mCRPC diagnosis (28).

Several other biomarkers can also be found as factors in multivariable analysis, improving the prognostic model performance. Besides the previously mentioned biomarkers, such as PSA, PSA kinetic, and inflammatory response cells, the other biomarkers can be divided into several categories. Those are biomarkers and factors specifically related to PC, such as the level of testosterone, duration of ADT before mCRPC diagnosis, and localization of metastasis sides (nodes, bones, visceral). General cancer-related biomarkers are represented by alkaline phosphatase (ALP) – an indicator of bone metastatic tumor load (29), lactate dehydrogenase (LDH) – an increased biomarker of highly proliferating cancer cells connected with enhanced glycolysis (30), and presence of CTCs (31, 32). In the last category, other health-related features, such as hemoglobin level, albumin level, or performance status, can be found. All these factors have been repeatedly used to boost the stratification significance for patients diagnosed with mCRPC (33–35). Several combinations and their prognostic model performance are shown in **Table 1**. Nevertheless, despite the depth of the current understanding of multivariable prognostic indicators, none have been accepted as a tool in clinical practice.

Beyond the general biomarkers listed above, a particular category related to neuroendocrine phenotype related to poor prognosis must be highlighted. Typically, the histologic feature of *de novo*-diagnosed patients is prostate adenocarcinoma. However, in some cases, neuroendocrine differentiation of the primary adenocarcinoma histology can be detected during treatment (36). So far, two mechanisms have been suggested for the emergence of this very aggressive form of PC. The first is based on the fact that neuroendocrine cells can be sparsely distributed in the original adenocarcinoma cells. Since the malignant neuroendocrine cells are not sensitive to androgen inhibition, they can start to grow during hormonal therapy and cause resistance to treatment (clonal expansion) (37). The second mechanism, transdifferentiation of adenocarcinoma cells to neuroendocrine prostate cancer, is often observed after androgen deprivation and other stress stimuli (38, 39). Still, this highly complex process can also be related to some genomic alterations (PTEN, TP53, RB1, epigenetic events) (40, 41). Notably, the proliferation of neuroendocrine cells will be reflected in an increased expression of typical neuroendocrine biomarkers such as chromogranin A (CgA), enolase 2 or immunohistochemistry markers (42). Therefore, these biomarkers can be used as indicators of poor survival outcomes. Conteduca et al. (43) analyzed serum CgA levels and their dynamics during the course of therapy with enzalutamide. This retrospective study showed that mCRPC patients with CgA level above 360 ng/mL before enzalutamide treatment in the second line had much worse OS (3.4 months) than the patients with the initial CgA A level below 120 ng/mL (OS not reached for 12 months). Therefore, increased neuroendocrine biomarkers can, independently from PSA, predict a poor survival outcome. Suspicion of the development of an aggressive variant of prostate cancer with low PSA level can be also reflected in carcinoembryonic antigen (CEA) (44) or other clinical manifestations, including lytic bone metastases, bulky lymphadenopathy, exclusive visceral metastases along with short intervals of ADT response (45).

## 3 Circulating cell-free DNA and AR signaling

The circulating cell-free DNA (ccfDNA) molecules are predominantly random fragments with a length of about 180 base pairs formed during cell disintegration (46). Cancer cells contribute significantly to ccfDNA levels in the blood, leading to a higher abundance of cancer-derived ccfDNA. Healthy individuals typically have about 1-10 ng of ccfDNAs in one mL of blood (47, 48) and those levels are consistently elevated in cancer patients (49–51). Despite not being intact, ccfDNA molecules are still valuable for predictive and diagnostic purposes. Specific assays can examine different gene changes, such as point mutations, deletions, or amplifications (46). Furthermore, the amount of tumor-originated ccfDNA in plasma has been suggested as an independent prognostic biomarker for CRPC, in particular in combination with PSA evaluation (52–54). For more in-depth information on ccfDNA, dedicated reviews are available (48, 55–60).

Androgen receptor (AR) primarily drives PC proliferation and disease progression, and its signaling pathway is targeted by both ADT and ARPI. PC can develop various types of resistance, some of which are based on genetic or proteomic modifications of the AR (61, 62). The AR gene can be amplified (copy-number variation, CNV) or altered by point mutations primarily occurring in the ligand binding site (63–65). Approximately 60% of mCRPC samples exhibit aberrations in the AR gene (66), with nearly 50% of mCRPC cases showing AR gene amplification (67, 68). The plasma samples of CRPC and hormonal-sensitive patients were examined for AR CNV. However, CNV of AR was found exclusively in CRPC patients (69). AR gene amplification was associated with worse OS and/or PFS in both ADT and ARPI-treated patients (70–73). Later, CNV in different regions of the AR gene was found in 38% of mCRPC samples by Du et al. in 2020. They also suggested the assessment of CNV in the AR enhancer region and CNV in exon 8 of the AR gene as an excellent prognostic marker. The AR enhancer is a region 650 kb upstream of AR that contributes to the progression of mCRPC (69, 74, 75). The AR and its enhancer were also analyzed by targeted sequencing, which found alterations in 45% of metastatic patients (76). Importantly, this assay revealed amplification of AR/enhancer in 78% of patients with resistance to AR-directed therapy.

The AR gene point mutations are present in 15-20% of CRPC cases (68, 77). The missense AR mutations, including H875Y, T878A, and F876L, were detected in 18% of 62 CRPC patients treated with abiraterone (n=29), enzalutamide (n=19) or other agents (n=14) (73). The mutations H875Y and L702H were described in 11% of post-docetaxel, but in no chemotherapy-naïve, abiraterone-treated CRPC patients (71). Moreover, the presence of AR gene mutations H875Y and L702H was associated with worse OS in patients treated with abiraterone, or enzalutamide (71). The abiraterone-treated CRPC patients with AR aberration (mutations L702H or T878A or CNV of AR) had a higher risk of progression or primary resistance to abiraterone (70), and a significantly shorter PFS when AR aberrations (CNV of AR or mutations H875Y, L702H, and T878A) were analyzed (78). Interestingly, the presence of AR gene aberrations was not in correlation with the PFS of enzalutamide-treated CRPC patients in the same study (78). Importantly, whole genome sequencing of clinical samples showed a dramatically increased rate of AR binding sites which contributes to the upregulation of target genes and cancer progression (79). The AR alterations in relationship to PC progression and therapy are summarised in **Table 2**.

**Table 2:** The ccfDNA AR markers from plasma useful for prognostic stratification of CRPC (castration-resistant prostate cancer).

marker	cohort	analytic approach	outcome	reference
AR amplification	274 samples from 97 CRPC abiraterone-treated patients	sequencing	worse OS and PFS, primary resistance to abiraterone	Romanel et al., 2015 (70)
AR amplification	98 post-docetaxel CRPC ARPI treated patients, plasma 94 chemotherapy-naı ¨ve ARPI treated patients	digital PCR	worse OS in post-docetaxel-treated patients	Conteduca et al., 2017 (71)
AR amplification	70 prechemotherapy mCRPC patients on ADT	digital PCR	worse OS	Kohli et al., 2018 (72)
AR amplification, AR mutations H875Y and T878A	62 CRPC patients treated with ARPI or other agents	sequencing, genomic hybridization	worse PFS, ARPI resistance	Azad et al., 2015 (73)
AR mutation L702H and T878A	274 samples from 97 CRPC abiraterone-treated patients	sequencing	worse OS and PFS, primary resistance to abiraterone	Romanel et al., 2015 (70)
AR mutation L702H and T878A	98 post-docetaxel CRPC ARPI treated patients, plasma 94 chemotherapy-naı ¨ve ARPI treated patients	digital PCR	worse OS in post-docetaxel treated patients	Conteduca et al., 2017 (71)
AR amplification or mutation L702H, W742C, W742L, H875Y and T878A	102 CRPC patients treated by ARPI	digital PCR	poor response to abiraterone, but not to enzalutamide	Sumiyoshi et al., 2019 (78)
amplification of exon 8 of AR in combination with amplification of AR enhancer	108 mCRPC patients	digital PCR	worse OS	Du et al., 2020 (74)
amplification of AR enhancer	40 mPC ARPI treated patients	sequencing	worse OS and PFS	Dang et al., 2020 (76)

AR (androgen receptor), ARPI (AR-pathway inhibitors), PFS (progression free survival), OS (overall survival).

## 4 Methylation markers in CRPC

Apart from DNA alterations of the AR gene, the DNA methylation landscape of PC has gained interest as a potential marker for PC progression. Notably, ccfDNA extracted from CRPC patients’ plasma contains approximately 64% of tumor-specific DNA methylation patterns, making it suitable for investigating epigenetic changes in PC (80). In cases of PC progression, there is a notable increase in the levels of methylation in genes GSTP1, RASSF1A, APC, and RARB compared to patients without progression (81). The loss of GSTP1, RASSF1A, APC, and RARB expression due to methylation is observed even in the early stages of tumorigenesis as they serve as tumor suppressors involved in DNA repair, cell adhesion, cell cycle control, and signal transduction (82–84). Hypermethylation of GSTP1 and APC genes has also been elevated in the plasma of CRPC patients and was correlated with shorter OS (85). Additionally, the methylation of at least one gene among GSTP1, RASSF1a, APC, PTGS2, or MDR1 within plasma ccfDNA was associated with poor mCRPC patient outcomes (86). Besides the genes mentioned above, the hypermethylation of MDR1 may contribute to PC proliferation and drug resistance, while PTGS2 silencing was associated with an elevated chance of PC recurrence (87, 88).

Two other studies have identified methylation markers of poor OS. Methylation occurring in the promoter region of the cadherin 13 (CDH13) gene correlated with worse survival and elevated risk of death in serum samples from primary PC patients compared to age-matched controls (89). Notably, levels of methylated GSTP1 gene were detectable in 458 (81%) of mCRPC patients before undergoing docetaxel treatment, and these levels were associated with worse OS. Additionally, patients exhibiting methylated GSTP1 levels after two chemotherapy cycles experienced worsened OS and a shorter time to PSA progression (90). Moreover, a distinct methylation signature within the genes AKR1B1, LDAH, and KLF8 was identified as predictive of therapy failure in mCRPC patients’ plasma samples (91). The molecular consequences of silencing the genes mentioned above in PC prognosis and prediction of therapy response have yet to be clearly explained. Some clues may be found for KLF8 and LDAH. The KLF8 enhances the transcriptional activity of AR (92) and its methylation in therapy-resistant PC may indicate androgen-independent growth. Loss of LDAH, known as lipid droplet-associated hydrolase, has already been associated with prostate tumorigenesis (93). However, the molecular association between LDAH silencing, lipid metabolism, and driving cancer pathways has not been described yet.

The ccfDNA methylation of DOCK2, HAPLN3, and FBXO30 genes correlated with a shorter time to progression and CRPC occurrence and could be used for the identification of patients who could benefit from intensified treatment (94). While cytosine conversion to 5-methylcytosine commonly results in transcriptional repression, further conversion to 5-hydroxymethylcytosine (5hmC) is associated with transcriptional activation. Importantly, recent targeted sequencing of cell-free DNA has found 5hmC activation marker of proliferation TOP2A, and a marker of cell invasion and angiogenesis EZH2 in patients with an aggressive subtype of metastatic disease (95–97). TOP2A is an essential nuclear enzyme required to resolve topological stress associated with DNA replication (98). Its upregulation may induce rearrangements of genes that contribute to a more invasive phenotype. It has also been reported to enhance androgen receptor signaling by facilitating the transcription of androgen-responsive genes (99). EZH2, the catalytic subunit of the polycomb repressive complex 2, works in concert with histone deacetylases as epigenetic modifiers (100). Dual upregulation of TOP2A and EZH2 has also been associated with recurrence after prostatectomy as well as radiotherapy (101, 102).

The new generation sequencing analysis of ccfDNA methylome from CRPC plasma identified enrichment of AR binding sequences and hypomethylation of putative AR binding sites associated with the amplification of the AR gene and a more aggressive clinical course (103). Interestingly, the trimethylation of histone H3 lysine 27 (H3K27me3) has been implicated as an epigenetic marker potentially linked to prostate carcinogenesis (104). A significant decrease in H3K27me3 levels was found in the plasma of metastatic PC patients compared to those with localized or locally advanced PC (105). The ccfDNA methylation markers of PC progression are summarised in **Table 3**.

**Table 3:** ccfDNA methylation markers with connection to PC (prostate cancer) progression.

marker	cohort	analytic approach	outcome	reference
APC, GSTP1, RARB, and RASSF1A	42 PC patients on castration (20 patients developed CRPC), whole blood	methylation-specific PCR	progression of PC, CRPC occurrence	Rouprêt et al., 2008 (81)
APC, GSTP1, MDR1, PTGS2, or RASSF1a	76 CRPC patients, serum	methylation-specific PCR	poor therapy outcome	Okegawa et al., 2010 (86)
GSTP1 and APC	47 CRPC patients, plasma	methylation-specific PCR	correlates with shorter OS	Hendriks et al., 2018 (85)
GSTP1	562 mCRPC patients before docetaxel treatment, serum	methylation-specific PCR	worse OS, shorter time to progression	Mahon et al., 2019 (90)
tri-methylation of H3K27	22 local, 11 local advanced and 28 metastatic PC patients, plasma	ELISA-based	discriminate mPC from localized and advanced PC	Deligezer et al., 2010 (105)
CDH13 promoter	98 PC patients, serum	methylation-specific PCR	worse survival, elevated risk of death	Wang et al., 2014 (89)
DOCK2, HAPLN3, and/or FBXO30	102 localized PC patients 65 mCRPC patients, plasma	methylation-specific digital PCR	shorter time to progression and mCRPC occurrence	Bjerre et al., 2020 (94)
hypomethylation of putative AR binding sites	25 mCRPC ARPI treated patients, plasma	whole-genome sequencing	more aggressive PC	Wu et al., 2020 (103)
AKR1B1, LDAH, and KLF8	29 mCRPC, plasma	methylation-specific PCR	therapy failure prediction	Dillinger et al., 2022 (91)
5-hydroxymethylcytosine activation of TOP2A and EZH2	64 mCRPC, plasma	whole-genome sequencing	detection of aggressive PC phenotype	Sjostrom et al., 2022 (95)

CRPC (castration-resistant prostate cancer), ELISA (enzyme-linked Immunosorbent assay), mCRPC (metastatic castration-resistant prostate cancer), PCR (polymerase chain reaction), OS (overall survival).

## 5 Messenger RNA of AR and its splice variants

PC cells can develop different splice variants of AR (ARv) during resistance development (106). There are more than 20 known ARv (107), and they mostly do not contain a ligand binding domain (LBS), which is replaced by a variant-specific short peptide (108). The ARv7 has received the most attention as it is the most abundant ARv in PC (109) and contributes to PC growth in a low-androgen environment (110, 111). The connection between ARv7 expression and the highly aggressive PC was investigated in many studies, mostly with the use of CTCs (76, 112–116).

The usefulness of ARv7 as a marker of CRPC progression was discussed in two meta-analyses in 2020 with the same results. The ARv7 significantly correlated with the worst outcome in mCRPC patients treated with hormonal therapy (the worst OS, PFS, and PSA-PFS) or chemotherapy (OS, and PFS) with the use of data from 13 studies (117) or data from 21 studies (118). These findings were further supported by the recent meta-analysis by Khan et al., where the presence of ARv7 significantly correlated with shorter OS, PFS, and PSA-PFS in the whole CRPC-patients’ group as well as in ARPI-treated CRPC group (with a hazard ratio of 4.34). Need to note the majority of the 37 studies analyzed ARv7 in CTCs, and only 7 used whole blood or exosomal RNA (119). For instance, Qu et al. correlated ARv7 and PSA ccfRNA levels in the plasma of 81 abiraterone- and 51 enzalutamide-treated CRPC patients (120). All patients with higher ARv7 transcript levels had a shorter time to treatment failure. On the other hand, when considering multiple factors together in a multivariate model, significant results were only observed in the enzalutamide-treated patients. In a subsequent study conducted by Del Re et al. in 2019, full-length AR and ARv7 were analyzed in plasma samples from 73 CRPC patients before ARPI treatment using digital droplet PCR. They were able to identify full-length AR in all samples and ARv7 in 22% of samples. Moreover, a high number of AR copies (≥ 900 copies/mL) in plasma correlated with shorter PFS and OS. ARv7 showed an even better predictive value (121). A study by Stupolyte and colleagues evaluated the second most common ARv in PC samples ARv1 (108), which was present in 17% of CRPC plasma samples. Both elevated AR levels and the presence of ARv1 were associated with shorter PFS and OS (122). The ccf mRNA markers of CRPC progression are summarised in **Table 4**.

**Table 4:** ccfRNA markers of CRPC (castration-resistant prostate cancer) progression.

marker	cohort	analytic approach	outcome	reference
ARv7	36 CRPC patients, plasma	digital PCR	shorter OS and PFS	Del Re at al., 2017 (164)
ARv7	85 mCRPC patients before ARPI treatment, whole blood	digital PCR	high expression corelated with shorter PFS and OS	Seitz et al., 2017 (165)
ARv7	81 abiraterone and 51 enzalutamide treated CRPC patient, peripheral blood mononuclear cell fraction	digital PCR	shorter time to treatment failure	Qu et al., 2017 (120)
ARv7, HOXB13 and KLK2	37 mCRPC patients treated with abiraterone, whole blood	quantitative PCR	expression levels corelated with PFS and OS	Todenhöfer et al., 2017 (166)
ARv7	73 CRPC patients, plasma	digital PCR	prediction of OS and PFS	Del Re at al., 2019 (121)
full-length AR ≥ 900 copies/mL	73 CRPC patients, plasma	digital PCR	shorter OS and PFS	Del Re at al., 2019 (121)
ARv7, GRHL2, HOXB13, and FOXA1	115 mCRPC patients, whole blood	quantitative PCR	shorter OS, stratification of patients	Kwan et al., 2019 (167)
high level of full- length AR and/or ARv1	102 CRPC patients, whole blood	quantitative PCR	shorter OS and PFS	Stuopelyte et al., 2020 (122)

AR (androgen receptor), ARV7 (androgen receptor variant 7), ARPI (AR-pathway inhibitors), mCRPC (metastatic castration-resistant prostate cancer), PCR (polymerase chain reaction), PFS (progression free survival), OS (overall survival).

## 6 Non-coding ccfRNAs

The non-coding RNAs can be categorized based on their length into small non-coding RNAs (micro-RNAs, miRNAs) and long non-coding RNAs (lncRNAs). Plasma and serum samples can be utilized with comparable results for detecting miRNAs. Moreover, miRNAs demonstrate stability in the blood due to protection from endogenous RNAse activity (123). Several studies have provided insights into the possible role of miRNAs in the PC progression and development of CRPC (124–127).

The significance of miR-375 was demonstrated by several studies. The expression of miR-141 and miR-375 correlated with positive lymph node status and Gleason score in 71 samples of mCRPC (128). The upregulated miR-141, miR-375, and miR-378 were described in serum from CRPC patients, while miR-409-3p was significantly underexpressed compared to serum from low-risk localized patients (129).

Furthermore, the levels of miR-141 and miR-375 were associated with the docetaxel and ARPI treatment outcome in mCRPC patients. Levels of four miRNAs decreased after therapy started. However, levels of miR-141 and miR-375 elevated again at the time of radiological progression, showing potential to be markers of therapy failure and PC progression (130). Additionally, the miR-375 levels predicted which patients would develop metastases with 50% sensitivity, and 76% specificity (131) and were associated with progression-free survival in mCRPC patients treated with enzalutamide (132). The miR-375 can also be associated with non-cancerous diagnoses, e.g. diabetes (133–135). Further investigation is required to understand the impact of lifestyle, diet, and even circadian rhythms on cell-free molecules release into the bloodstream.

The patients with high expression of miR-7 and miR-221 in whole peripheral blood samples had shorter time to CRPC development. They had shorter OS than patients with low expression of these miRNAs. Therefore, the authors suggested the use of miR-7 for the prediction of CRPC occurrence (136). In the study by Lin et al., 2014, they analyzed 96 CRPC docetaxel-treated patients’ blood or plasma samples by a custom Taqman Array for 46 candidate miRNAs. While specific prognostic miRNAs were not identified, a combination of pre-docetaxel miR-200b levels, post-docetaxel miR-20a levels, pre-docetaxel hemoglobin levels, and visceral metastasis proved to be independent predictors of OS when used together.

In 2013, Watahiki et al. described 63 upregulated plasma miRNAs and four downregulated miRNAs in mCRPC compared to localized PC. They could distinguish between mCRPC and localized PC with higher specificity and sensitivity with the use of a specific combination of miRNAs rather than with the use of one miRNA. One set consisted of miR-141, miR-375 and miR-200c, the second included miR-151-3p, miR-423-3p, miR-126, miR-152 and miR-2, and the last miR-16 and miR-205. There were three most important molecules miR-141, miR-151-3p, and miR-16 from each group that increased the sensitivity of the PSA test and could be used for discrimination between localized PC and mCRPC (138). While these findings show promise, validation on a large cohort of patients is still required for routine use in personalized medicine. The prognostic miRNAs are summarized in **Table 5**.

**Table 5:** The summary of miRNAs useful for prognostic stratification of CRPC patients analysed by quantitative polymerase chain reaction.

marker	change in levels	cohort	outcome	reference
miR-20a miR-200b	downregulated upregulated	96 whole blood/plasma samples	shorter OS on docetaxel treatment	Lin et al., 2014 (137)
miR-7 miR-221	upregulated upregulated	45 whole blood samples	correlation with the time to CRPC and progression	Santos et al., 2014 (136)
miR-182-5p miR-375	upregulated	252 plasma samples	more advanced pathologic stage, prediction of the metastasis development	Bidarra et al., 2019 (131)
miR-375 miR-3687	upregulated	40 whole blood samples	reflect progression-free survival after enzalutamide treatment	Benoist et al., 2020 (132)
miR-141 miR-375	upregulated	71 serum samples	positive lymph-node status and Gleason score	Brase et al., 2011 (128)
miR-141 miR-375 miR-378 miR-409-3p	upregulated upregulated upregulated downregulated	84 serum samples	differentiation between CRPC and localized PC	Nguyen et al., 2013 (129)
miR-141 miR-375	upregulated	84 plasma samples	elevated baseline levels correlated with shorter OS, prediction of docetaxel or ARPI failure	Zedan et al., 2020 (130)

ARPI (AR-pathway inhibitors), OS (overall survival).

The role of lncRNAs in PC development and therapy resistance has been gradually unveiled (139–147). The prostate cancer antigen 3 (PCA3) is one of the most prostate-specific biomarkers routinely used in urine examination. Notably, PCA3 was also analyzed using a digital PCR approach in plasma LB samples from 201 PC patients. It was combined with other lncRNAs, S100A4 and MRC2, as a minimally invasive biomarker for screening and aggressiveness stratification of PC patients (148). Additionally, the lncRNA TUC338 was associated with shorter OS in a cohort of 52 PC patients (142). The H19 lncRNA encapsulated in EVs was significantly elevated in ARPI-resistant CRPC patients, however, the study was conducted on a small number of patients (only 6 ARPI-resistant CRPC patients) (149). In general, lncRNAs are frequently utilized as diagnostic biomarkers for PC when examining tissues (150). Despite the efforts of researchers in the field of lncRNAs, the implementation of circulating lncRNAs as LB markers for CRPC progression monitoring has yet to be realized.

## 7 Emerging role of circulating lipids in CRPC monitoring

Over the past decade, many studies have demonstrated the significance of lipid metabolism in PC development and progression (151, 152). We have shown that cholesterol metabolism is essential for the intratumoral production of androgens (153, 154), which is in line with clinical observation that medication with statins prolongs the time to progression in patients on ADT (153, 154). Furthermore, targeting sphingosine kinase, an enzyme involved in lipid metabolism, has emerged as a potential therapeutic approach to overcome enzalutamide resistance and improve patient treatment outcomes (155).

Several studies highlighted the usefulness of lipid detection in LB samples for prognostic purposes. A distinct plasma signature containing two sphingolipids (ceramide d18:1/24:1 and sphingomyelin d18:2/16:0) and glycerophospholipid phosphatidylcholine 16:0/16:0 was associated with poor prognosis and OS of mCRPC patients (156). The aberrations in circulating ceramide levels were associated with poor clinical outcomes in both localized and metastatic PC (157), prompting further studies to explore the combined effects of lipidomic and genetic aberrations on clinical outcomes in mCRPC. The elevated levels of the three-lipid signature and abnormalities in one or more genes (AR, TP53, RB1, or PI3K) were associated with worse prognoses in mCRPC patients (158). The same group has recently introduced a modified circulating lipid biomarker signature (PCPro), which contains ceramides (d18:1/18:0), (d18:1/24:0), and (d18:1/24:1), triglycerides, and total cholesterol. PCPro may be a clinically accessible panel of blood lipid markers capable of prospectively identifying men with mCRPC with a poor prognosis (159).

## 8 Conclusion

This article summarizes recent knowledge about liquid biopsy markers suitable for monitoring CRPC progression. Especially promising are AR alterations, miR-375, methylation markers, and emerging lipidomic analyses. These markers are discussed alongside standard clinical parameters, such as PSA, LDH, or CRP. Mutational and methylation analyses provide cancer-specific information from relatively stable ccfDNA; however, the methods may be laborious and expensive. Expression analysis may be hampered by mRNA degradation, but short miRNAs have repeatedly been shown to be reliable biomarkers. Lipidomics open new avenues in cancer monitoring thanks to fast mass spectrometry analysis. The standard clinical parameters should always serve as benchmarks in new biomarker studies.

Integrating genomic, transcriptomic, and epigenomic biomarkers in clinical practice can potentially revolutionize the management of CRPC. Among the various biomarkers studied, AR alterations, including gene amplifications, point mutations, and the AR enhancer region CNV, stand out as having significant prognostic and predictive value (70–78). Specifically, the AR enhancer CNV has been suggested as an excellent prognostic marker due to its strong association with progression in mCRPC and resistance to AR-directed therapy (76).

Additionally, ccfDNA methylation and ccfRNA markers, such as ARv7, have shown potential in providing prognostic information. When analyzed from non-invasive liquid biopsies, methylation markers could offer insights into tumor biology and patient prognosis (80, 81, 85, 86, 89–91, 94, 95, 103, 105). ARv7, a splice variant of the AR, has been linked to resistance to AR-targeted therapies, making it a promising predictive biomarker for therapy response (117–121). Early detection of treatment failure followed by the switch of therapy may significantly improve the patient´s prognosis.

Despite these advancements, it is crucial to standardize sample acquisition and validation methods across laboratories to ensure reproducibility and accuracy. Future research should focus on validating these biomarkers in large, independent cohorts and developing robust, clinically applicable assays. Adhering strictly to standardized sample collection procedures and employing robust methods are essential prerequisites for reproducing and facilitating the clinical use of LB markers. Their integration into routine clinical practice could improve prognostic stratification and personalized treatment strategies for patients with CRPC. After proper and multicentric validation, these promising biomarkers have great potential to enhance the care of metastatic prostate cancer patients.
